# Knockdown of carnitine palmitoyltransferase I (CPT1) reduces fat body lipid mobilization and resistance to starvation in the insect vector *Rhodnius prolixus*

**DOI:** 10.3389/fphys.2023.1201670

**Published:** 2023-07-04

**Authors:** Iron F. De Paula, Samara Santos-Araujo, David Majerowicz, Isabela Ramos, Katia C. Gondim

**Affiliations:** ^1^ Instituto de Bioquímica Médica Leopoldo de Meis, Universidade Federal do Rio de Janeiro, Rio de Janeiro, Brazil; ^2^ Departamento de Biotecnologia Farmacêutica, Faculdade de Farmácia, Universidade Federal do Rio de Janeiro, Rio de Janeiro, Brazil; ^3^ Programa de Pós-Graduação em Biociências, Universidade do Estado do Rio de Janeiro, Rio de Janeiro, Brazil

**Keywords:** carnitine palmitoyltransferase I (CPT1), β-oxidation, starvation, lipid droplet, *Rhodnius prolixus*

## Abstract

The energy stored in fatty acids is essential for several critical activities of insects, such as embryogenesis, oviposition, and flight. *Rhodnius prolixus* is an obligatory hematophagous hemipteran and vector of Chagas disease, and it feeds infrequently on very large blood meals. As digestion slowly occurs, lipids are synthesized and accumulate in the fat body, mainly as triacylglycerol, in lipid droplets. Between feeding bouts, proper mobilization and oxidation of stored lipids are crucial for survival, and released fatty acids are oxidized by mitochondrial β-oxidation. Carnitine palmitoyl transferase I (CPT1) is the enzyme that catalyzes the first reaction of the carnitine shuttle, where the activated fatty acid, acyl-CoA, is converted to acyl-carnitine to be transported into the mitochondria. Here, we investigated the role of CPT1 in lipid metabolism and in resistance to starvation in *Rhodnius prolixus*. The expression of the CPT1 gene (*RhoprCpt1*) was determined in the organs of adult females on the fourth day after a blood meal, and the flight muscle showed higher expression levels than the ovary, fat body, and anterior and posterior midgut. *RhoprCpt1* expression in the fat body dramatically decreased after feeding, and started to increase again 10 days later, but no changes were observed in the flight muscle. β-oxidation rates were determined in flight muscle and fat body homogenates with the use of ^3^H-palmitate, and in unfed females, they were higher in the flight muscle. In the fat body, lipid oxidation activity did not show any variation before or at different days after feeding, and was not affected by the presence of etomoxir or malonyl-CoA. We used RNAi and generated RhoprCPT1-deficient insects, which surprisingly did not show a decrease in measured ^3^H-palmitate oxidation rates. However, the RNAi-knockdown females presented increased amounts of triacylglycerol and larger lipid droplets in the fat body, but not in the flight muscle. When subjected to starvation, these insects had a shorter lifespan. These results indicated that the inhibition of *RhoprCpt1* expression compromised lipid mobilization and affected resistance to starvation.

## Introduction

The energy stored in fatty acids is important for several critical activities of insects, such as embryogenesis, oviposition, and flight. However, to access this energy, these fatty acids need to be broken down in a pathway that makes maximum use of this energy by channeling it toward ATP production in the mitochondria. This pathway is mitochondrial β-oxidation. This pathway is well described in mammals, and the enzyme carnitine palmitoyltransferase I (CPT1), which is part of the carnitine shuttle and responsible for the formation of acyl-carnitine moieties from acyl-CoA species, plays a central role in β-oxidation. CPT1 is located on the exterior face of the mitochondria, and performs the limiting step of the pathway (Houten and Wanders, 2010). There are three different isoforms in mammals, CPT1A, CPT1B, and CPT1C, each with its own particular characteristics regarding expression in tissues and physiologic function (Price et al., 2002; Luiken et al., 2009). Insect models were of great importance in the past for understanding the β-oxidation pathway, with the demonstration that *Tenebrio molitor* requires an essential growth factor, which was initially called vitamin B_t_ but was later revealed to be carnitine, a substrate of CPT1 (Fraenkel, 1948; Carter et al., 1952). Despite this important first contribution, information about this pathway in insects is scarce.

Unlike mammals, insects and other arthropods have only one isoform of CPT1 (Lavárías et al., 2009; Price et al., 2010; Majerowicz et al., 2017). Insect development and adaptability to nutritional and environmental stresses both depend on the proper operation of the β-oxidation pathway. This is exemplified by the fact that *D. melanogaster withered* (*whd*) mutants, in which CPT1 is knocked out, are very sensitive to starvation (Strub et al., 2008). Additionally, the knockout of other components of the pathway downstream of CPT1 results in a reduction in life expectancy, deficiencies in locomotor activity, reduced oviposition and a reduced capacity of fatty acid oxidation in *D. melanogaster* (Kishita et al., 2012). However, some data indicate that CPT1 may not perform the rate-limiting step of the pathway, at least in some insects, such as the moths *Prodenia eridania* and *Amphion floridensis* (Stevenson, 1968; O’Brien and Suarez, 2001).

*R. prolixus* is a hematophagous insect with great economic and medical importance since it is one of the main vectors of Chagas disease in Central and South America (Antinori et al., 2017). Like other insects, it possesses most of the enzymes involved in β-oxidation, as shown by bioinformatic analyses (Majerowicz et al., 2017), and there are only a few studies concerning lipid oxidation in this model. The last three reactions of this pathway are catalyzed by the mitochondrial trifunctional protein (MTP), and the inhibition of the gene expression of the alpha subunit (HADHA, hydroxyacyl-CoA dehydrogenase trifunctional multienzyme complex subunit alpha) in *R. prolixus* impairs lipid mobilization during starvation and results in higher contents of triacylglycerol (TG) and larger lipid droplets (LDs) in the fat body, where lipids are stored after a blood meal (Arêdes et al., 2022). In addition, oviposition is decreased in these insects, as is the flight capacity. The knockdown of RhoprACSL2, a long-chain acyl-CoA synthetase that is one out of two isoforms responsible for the conversion of fatty acids to acyl-CoA moieties in this insect, also decreases oviposition and leads to a 90% reduction in β-oxidation rates, indicating that this enzyme activates fatty acids channeled for β-oxidation in *R. prolixus* (Alves-Bezerra et al., 2016)*.* These results show that the proper activity of mitochondrial fatty acid oxidation is critical for reproductive success. On the other hand, the knockdown of RhoprGPAT1, a glycerol-3-phosphate acyltransferase that catalyzes the first step of *de novo* TG synthesis, leads to a 2-fold increase in β-oxidation rates and a 65% decrease in TG content in the fat body (Alves-Bezerra, et al., 2017). Thus, when fatty acids are not efficiently directed to TG synthesis, they are oxidized, and reserves are not properly formed, showing that there is a fine balance between lipid synthesis and degradation.

Although the β-oxidation pathway is fundamental for the processes of reproduction and locomotor activity and to guarantee metabolic homeostasis, the role of the CPT1 ortholog of *R. prolixus* (RhoprCPT1) in adaptation to starvation is unclear. *R. prolixus* is an intermittent feeder, and weeks can go by between blood meals. Therefore, the proper mobilization of lipid reserves in the fat body between feeding bouts is critical for survival, and RhoprCPT1 likely plays a central role in this process. Failure to mobilize these reserves may have implications for survival, oviposition, and flight. Thus, as we previously identified its gene in the *R. prolixus* genome (Majerowicz et al., 2017), we investigated the role of RhoprCPT1 in insect physiology, especially under starvation conditions, by assessing its gene expression and β-oxidation biochemical activity. We found that after inhibiting *RhoprCpt1* gene expression using RNAi, survival during starvation and lipid mobilization were affected.

## Materials and methods

### Insects

Adult females of *R. prolixus* were kept in a colony at 28 ± 2°C, with relative humidity of 65%–85%, and a 12 h/12 h light and dark cycles. The experimental insects were adult females that fed on live rabbits in 3-week intervals, and were used either after the second or third blood meal (fed condition) or before feeding (unfed condition, 21 days after the last blood meal). All animal care and experimental protocols were conducted following the guidelines of the Committee for Evaluation of Animal Use for Research from the Federal University of Rio de Janeiro (CAUAP-UFRJ) (process number 01200.001568/2013-87, order number 149/19), and the NIH Guide for the Care and Use of Laboratory Animals (ISBN 0-309-05377-3).

### Phylogenetic analysis

The genomes of *R. prolixus* (Mesquita et al., 2015), *D. melanogaster* (Adams et al., 2000), the bee *Apis mellifera* (Weinstock et al., 2006), the postman butterfly *Heliconius melpomene* (Consortium, 2012), the beetle *Tribolium castaneum* (Richards et al., 2008), the aphid *Acyrthosiphon pisum* (Richards et al., 2010), the whitefly *Bemisia tabaci* (Chen et al., 2019), the bed bug *Cimex letularius* (Rosenfeld et al., 2016), the termite *Zootermopsis nevadensis* (Terrapon et al., 2014), the louse *Pediculus humanus* (Kirkness et al., 2010), the nematode *Caenorhabditis elegans* (Consortium, 1998), and *Homo sapiens* (Nurk et al., 2022) were explored. All proteins containing the Pfam domain (Mistry et al., 2021) PF00755 (choline/carnitine o-acyltransferase domain) were obtained from the Ensembl genomes database (Yates et al., 2022) using the BioMart tool (Kinsella et al., 2011). The primary sequences were aligned with the Clustal W algorithm (Larkin et al., 2007), and phylogenetic analysis was performed by the maximum likelihood method (Felsenstein, 1981) with 500 bootstrap replicates in MEGA 11 software (Tamura et al., 2021). The dendrogram was visualized with the program FigTree v.1.4.4.

### Gene expression analysis

For gene expression analysis, anterior and posterior midguts, abdominal fat bodies, ovaries, and flight muscles were obtained from females on the fourth day after a blood meal. To analyze the time-dependent response, the fat bodies and flight muscles were dissected before feeding (day 0) and on different days after a blood meal. Total RNA was isolated from samples (3–5 organs), in TRIzol Reagent (Invitrogen, Carlsbad, CA, United States). After quantification with a NanoDrop Spectrophotometer (Thermo Fisher Scientific, Waltham, MA, United States), the integrity and quality of the RNA samples were analyzed by electrophoresis on a 2% agarose gel (UBS, Cleveland, OH, United States), and RNA was considered intact when the 18S rRNA band was observed. RNA samples (1 µg) were treated with RNase-free DNase I (Thermo Fisher Scientific) and used to synthesize cDNA with the High Capacity cDNA Reverse Transcription Kit (Applied Biosystems Inc., Foster City, United States).

Quantitative PCR (qPCR) was performed in a StepOne Real-Time PCR System (Applied Biosystems) using SYBR Green PCR Master Mix (Applied Biosystems) under the following conditions: 10 min at 95 °C, followed by 40 cycles of 15 s at 95 °C and 45 s at 60 °C. qPCR amplification was performed using specific primers for the target genes (Supplementary Table S1) designed using Primer3 software (Rozen and Skaletsky, 2000). *Rhopr18S* (for the fat body and comparisons between various organs) or *RhoprElf1* (for flight muscle) gene amplification was used for normalization (Majerowicz et al., 2011), and their amplification was constant under our experimental conditions. Amplification specificity analysis and qPCR controls to detect contamination were carried out following the MIQE guidelines (Bustin et al., 2009). The ΔΔCq values were calculated based on the Cq values obtained as described previously (Livak and Schmittgen, 2001) and were used for statistical analyses. Relative expression values (2^−ΔΔCq^) were used only for data plotting.

### Gene knockdown using double-stranded RNA (dsRNA)

Double stranded RNA (dsRNA) for the *RhoprCpt1* (dsCpt1) gene (VectorBase Gene ID: RPRC0056*39*; Majerowicz et al., 2017) was synthesized with the MEGAScript RNAi Kit (Thermo Fisher Scientific) using specific primers (Supplementary Table S1). Ten days after a blood meal, fed adult females were injected with 2 µg of dsRNA using a 10 μL microsyringe (Hamilton Company, Reno, NV, United States), and were dissected 11 days later on the 21st day after the blood meal (starvation condition). Knockdown efficiency was confirmed by qPCR. dsRNA for the bacterial *MalE* gene (GenBank ID: 948538) was used as a control (dsMal) (Hansen et al., 2005).

### Lifespan

Ten days after a blood meal, fed adult females were injected with dsRNA and were observed daily until all the insects had died.

### Determination of TG content

On day 21 after feeding (starvation condition), abdominal fat body and total flight muscle were dissected from dsRNA injected insects, and individually homogenized in phosphate buffered saline (PBS; 10 mM sodium phosphate buffer, pH 7.4, 0.15 M NaCl). The TG content was then determined with the Triglycerides 120 colorimetric kit (Doles Reagents, Goiânia, Brazil).

### Fatty acid oxidation assay

Fat bodies and/or flight muscles were obtained from five insects (unfed or on different days after a blood meal), washed in 0.15 M NaCl and homogenized in a Potter-Elvehjem homogenizer (30 strokes) in 200 μL of cold buffer H containing 10 mM Hepes-KOH, pH 7.4, 0.25 M sucrose, 1 mM ethylenediamine tetraacetic acid (EDTA), 1 mM dithiothreitol (DTT), and 0.002% v/v protease inhibitor cocktail (Sigma-Aldrich, Saint Louis, United States). The homogenates were then centrifuged at 1,000 *g* for 5 min, and the supernatants were collected. After protein concentration determination (Lowry et al., 1951), the supernatant (30 μg protein) was incubated with 8 μCi ^3^H-palmitate (0.1 μCi/μL; PerkinElmer Inc., Waltham, MA, United States) in the presence of 75 mM Tris–HCl, pH 7.4, 2 mM MgCl_2_, 2 mg/mL fatty acid free albumin, 5 mM ATP, 5 mM DTT, 0.2 mM coenzyme A, 10 mM L-carnitine, and 20 mM palmitate (200 μL final volume), at 28 °C for 30 min, as described previously (Alves-Bezerra et al., 2016; Alves-Bezerra et al., 2017). Reactions were stopped with 200 μL cold perchloric acid (24%), and blanks were performed with the addition of homogenates to an incubation medium already containing perchloric acid. The reaction mixtures were then incubated at 4 °C for 12-16 h to precipitate albumin-bound ^3^H-palmitate. Samples were centrifuged at 1,000 *g* at 4 °C for 5 min, supernatants were collected and lipids were extracted into chloroform (Bligh and Dyer, 1959). The radioactivity present in the oxidized products in the aqueous phase (100 µL) was measured using a liquid scintillation counter. To test the effects of etomoxir or malonyl-CoA, samples were incubated as described above, in the presence of increasing concentrations of these substances (Sigma-Aldrich) after a 10 min preincubation period.

### Nile Red staining of lipid droplets

Fat bodies were obtained from dsRNA-injected insects on the 21st day after feeding (at least three females) and stained with Nile Red and DAPI, as previously described for *R. prolixus* LD analysis (Defferrari et al., 2016). The organs were incubated for 15 min in 1 mg/mL Nile Red (Sigma-Aldrich) and 2 mg/mL DAPI (Sigma-Aldrich) in 75% glycerol. Tissues were mounted in 100% glycerol and immediately imaged on a Leica TCS-SPE laser scanning confocal microscope, in two independent experiments. The excitation wavelengths used were 543 nm for Nile Red and 280 nm for DAPI, and the peripheral regions of the fat bodies were analyzed. The average diameters of the LDs were obtained from three images for each group using DAIME image analysis software after edge detection automatic segmentation (Daims et al., 2006). The LD diameters were plotted in a frequency histogram (bin width: 1).

### Statistical analyses

ΔΔCq mean values obtained from qPCR experiments were subjected to Grubb’s test to detect outliers (Burns et al., 2005), and the comparisons among different conditions were performed using one-way ANOVA followed by Tukey’s multiple comparison test. The relative expression values (2^−ΔΔCq^) were used only for graph construction. Differences in survival curves were analyzed using the log-rank test. Other results were analyzed by Student’s t-test for the comparison of two different conditions and one-way ANOVA followed by Tukey’s multiple comparison test for more than two conditions. All statistical analyses were performed using Prism 5.0 software (GraphPad Software, San Diego, United States), and differences were considered significant at *p* < 0.05.

## Results

### Phylogenetic analysis

**FIGURE 1:**
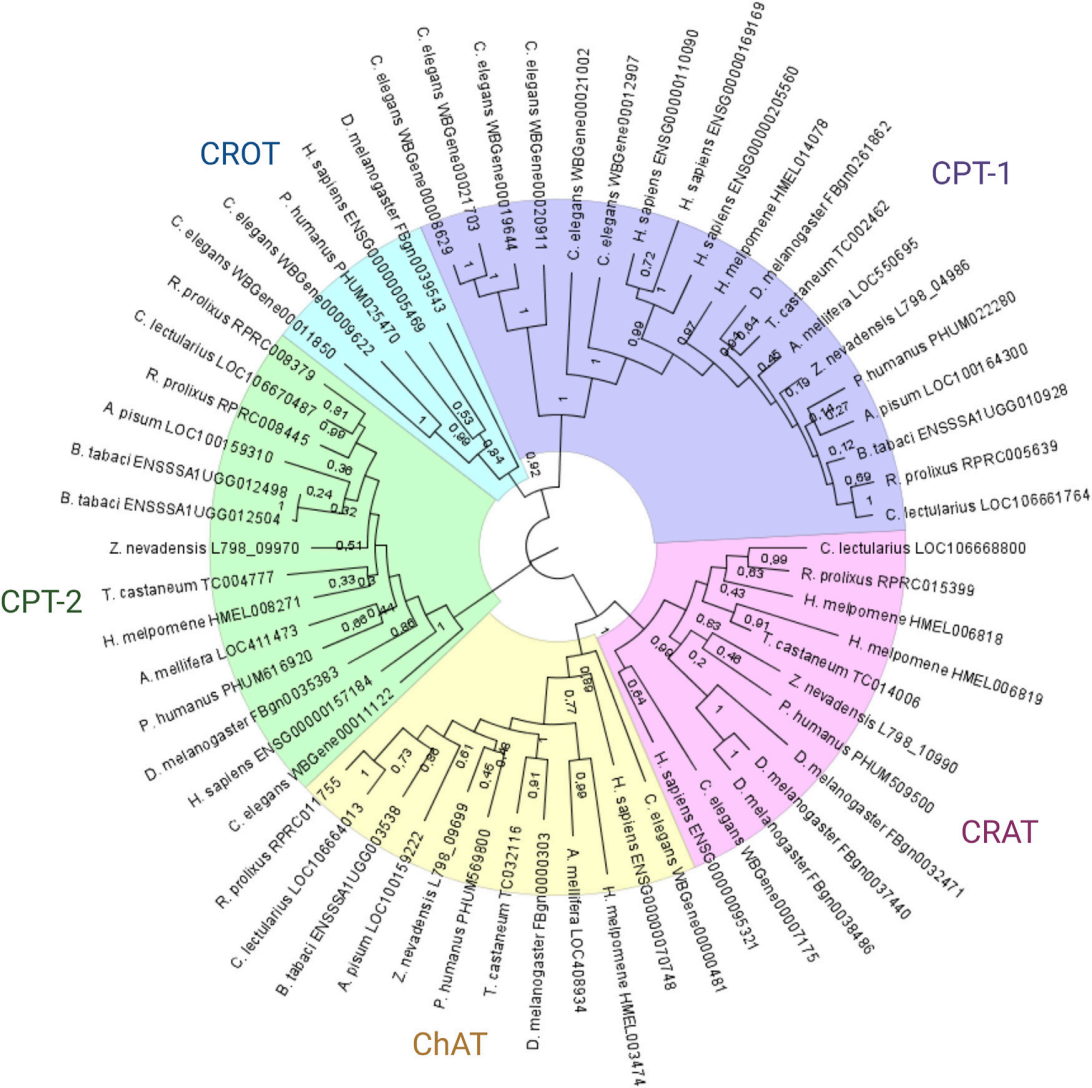
Phylogenetic analysis of *Rhodnius prolixus* choline/carnitine o-acyltransferase proteins. Protein sequences with Pfam domain PF00755 (choline/carnitine o-acyltransferase domain) from different species were aligned using ClustalW, and the dendrogram was constructed via the maximum likelihood method. Bootstrap values are indicated in branches, and the bars indicate substitutions per site. CRAT: carnitine o-acetyltransferase genes; CROT: carnitine o-octanoyl-transferase genes; CPT1: carnitine o-palmitoyltransferase 1 genes; CPT-2: carnitine o-palmitoyltransferase 2 genes; ChAT: choline o-acetyltransferase genes.

The phylogenetic analysis of proteins with the choline/carnitine o-acyltransferase domain identified five different groups of enzymes: CTP1 and 2, carnitine o-acetyltransferase (CRAT), choline o-acetyltransferase (ChAT) and carnitine o-octanoyl-transferase (CROT) (Figure 1). All genomes analyzed showed 1 to 1 orthologs in the CPT1 and ChAT groups. However, in the CPT2 group, the *R. prolixus* and *B. tabaci* genomes showed two paralogs of these genes. Interestingly, the tree architecture indicated that these duplications were independent, with the *B. tabaci* lineage duplication being more recent than that of *R. prolixus*. On the other hand, the CROT clade suffered extensive gene loss, and only *D. melanogaster* and *P. humanus* maintained a copy of this gene in their genome. Finally, the CRAT clade showed great diversity; while *D. melanogaster* had three paralogs and *H. melpomene* had two, both *A. pisum* and *B. tabaci* showed the loss of this gene.

The predicted structure of RhoprCPT1, containing 790 amino acids, possesses the canonical carnitine acyltransferase domain (PF 00755) (Supplementary Figure S1) used for the phylogenetic analysis (Figure 1); thus, it can be clearly classified as a CPT1 protein, closely related to other insect and mammalian CPT1 sequences. The CPT1s of the studied mammals have an N-terminal domain of approximately 160 residues that is required for protein insertion in the outer mitochondrial membrane and contains two transmembrane segments, which give the protein a bitopic topology in which both its N- and C-termini face the cytosol (Fraser et al., 1997; Cohen et al., 2001). These transmembrane sequences are also present in RhoprCPT1 (Supplementary Figure S1).

### *RhoprCpt1* gene expression patterns

**FIGURE 2:**
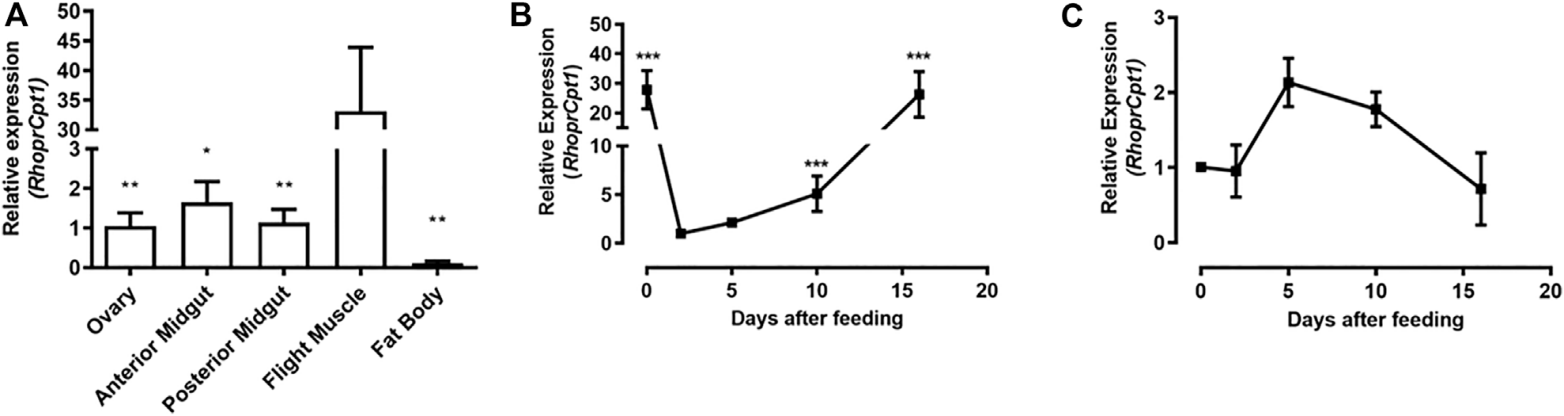
*RhoprCpt1* gene expression in *Rhodnius prolixus* organs. **(A)** Adult females were dissected on the fourth day after a blood meal, and their organs were harvested. Alternatively, the females were dissected before feeding (day 0) and on days 2, 5, 10, and 16 after a blood meal, and the **(B)** fat body and **(C)** flight muscle were collected. Total RNA was extracted from the samples and *RhoprCpt1* mRNA levels were quantified by qPCR using *Rhopr18S* or *RhoprElf1* expression as a reference. The results are means ± SEM (*n* = 3–5) and were analyzed by one-way ANOVA followed by Tukey’s multiple comparison post-test. In **(A)** (*) and (**): significantly different from the flight muscle at *p* < 0.05 and 0.01, respectively; in **(B)** (***): significantly different from day 2 at *p* < 0.001.

*RhoprCpt1* expression was measured in the ovary, anterior and posterior midgut, flight muscle and fat body, organs related to reproduction, digestion, locomotor activity, and lipid storage, respectively. On the fourth day after feeding, *RhoprCpt1* gene expression was detected in all the analyzed organs (Figure 2A). The flight muscle presented the highest relative levels of *RhoprCpt1* transcripts, with approximately 30 times higher relative abundance of transcripts than the anterior and posterior midguts and ovary. The fat body, despite being a central organ in insect lipid metabolism, showed a low level of *RhoprCpt1* gene expression, similar to the ovary and anterior or posterior midgut.

Since the fat body is the organ responsible for ensuring that the energetic needs of the insect are met in the intervals between blood meals, which are infrequent, we further investigated *RhoprCpt1* expression in this organ as well as in the flight muscle, which presented the highest gene expression level. As each blood meal triggers a chain of metabolic events and changes in the expression of diverse genes in *R. prolixus* (Leyria et al., 2020), we determined *RhoprCpt1* expression levels in the fat body before feeding and in the course of blood meal digestion (Figure 2B). The expression of *RhoprCpt1* significantly fluctuated, showing mRNA levels approximately 30 times higher in the unfed state than at 2 days after the blood meal. At 10 days after feeding, it was already higher than at day two, returning to unfed levels around the 15th day after the blood meal. The mRNA levels of *RhoprCpt1* in the flight muscle were stable during the same period (Figure 2C).

### Fatty acid β-oxidation activity

**FIGURE 3:**
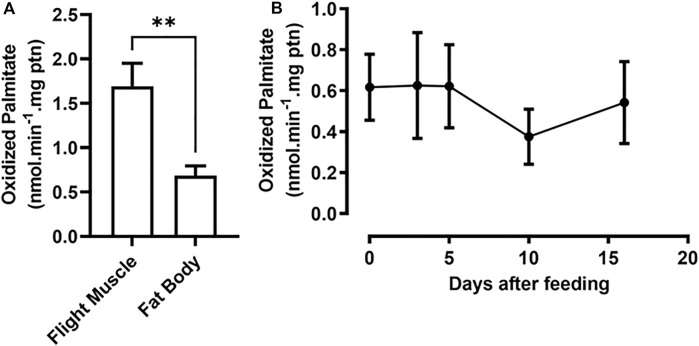
Fat body β-oxidation rates are constant before and after a blood meal. **(A)** Adult females were dissected before feeding (day 0) and their fat bodies and flight muscles were collected. **(B)** Females were also dissected before feeding and on different days after a blood meal (days 2, 5, 10, and 16), and their fat bodies were collected. After homogenization, the samples were used in β-oxidation assays, as described in Materials and Methods. The results are means ± SEM. **(A)** (**): significantly different at *p* < 0.01, by Student’s t-test, *n* = 3. **(B)** *p* > 0.05, by one-way ANOVA, *n* = 5.

β-Oxidation activity was measured in the fat body and flight muscle of unfed insects as a starting point for understanding the contribution of the pathway to insect metabolism. Flight muscles showed approximately 2.5 times the β-oxidation rates found in the fat body (Figure 3A). As the fat body is a central organ for lipid metabolism and energetic homeostasis, the β-oxidation rates were measured in this organ before feeding (day 0) and at various days after a blood meal, corresponding to different points in the digestive/reproductive cycle. Surprisingly, the observed β-oxidation rates were constant during the entire period, indicating that there was no modulation of the oxidation rates after the blood meal (Figure 3B), despite the observed differences in gene expression. As a control experiment, the assay was performed in the absence or presence of carnitine, as this compound is required for CPT1 activity in mitochondrial β-oxidation, and fatty acid oxidation was stimulated by carnitine addition (Supplementary Figure S2).

**FIGURE 4:**
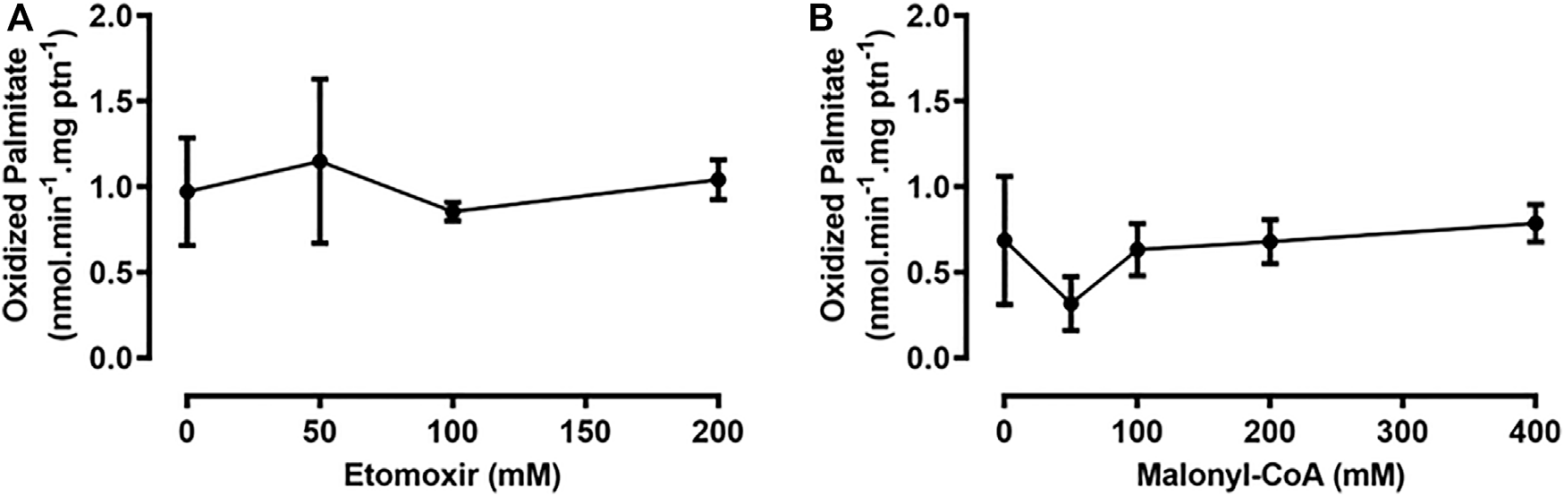
The addition of etomoxir and malonyl-CoA had no effect on β-oxidation in the fat body or flight muscle. Adult unfed females were dissected, and their fat bodies were collected, homogenized, and used for β-oxidation assays, in the presence of etomoxir **(A)** or malonyl-CoA **(B)**, after preincubation with these substances for 10 min. No statistically significant difference was observed, after analysis by one-way ANOVA (*p* > 0.05; *n* = 3).

Etomoxir is an irreversible inhibitor of the mammalian isoform CPT1A; however, it is not able to decrease CPT1B-dependent β-oxidation rates (Luiken et al., 2009; Ruffer et al., 2009). Hence, we set out to use etomoxir, as a pharmacological approach for inhibiting RhoprCPT1, which we would later use as a tool for studying the effects of such inhibition during starvation. However, when etomoxir was added to the assays with the fat body samples, it did not cause any reduction in β-oxidation rates (Figure 4A). Malonyl-CoA is the product of the carboxylation of acetyl-CoA by the enzyme acetyl-CoA carboxylase (ACC), the first step in *de novo* fatty acid synthesis (Ameer et al., 2014), and it acts as a potent inhibitor of mammalian β-oxidation in the liver (McGarry and Brown, 1997). However, despite being found in skeletal muscle, it does not show the same inhibitory effect on β-oxidation rates in that tissue (McGarry, 2001; Luiken et al., 2009). The addition of malonyl-CoA to the samples in the β-oxidation assay presented no effect on the measured rates in the fat body (Figure 4B). Thus, *R. prolixus* β-oxidation in these organs seems to be unaffected by these pharmacological and physiological inhibitors.

### Knockdown of *RhoprCpt1*

**FIGURE 5:**
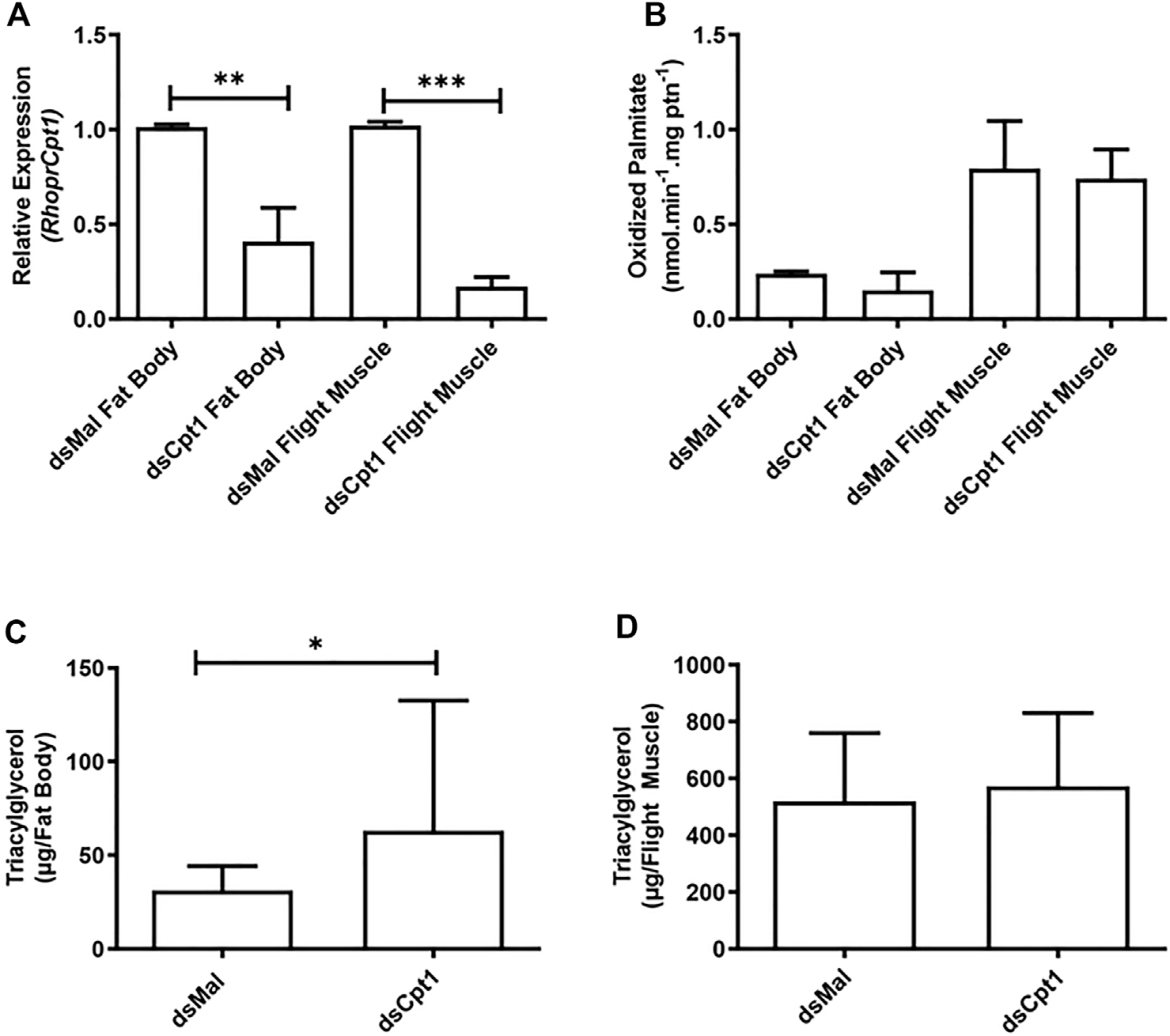
Knockdown of *RhoprCpt1* leads to TG accumulation in the fat body. On the 10th day after a blood meal, adult females were injected with 1 µg of dsRNA for *RhoprCpt1* or *Mal* (control), were dissected 11 days later (starvation condition; day 21), and the fat body and flight muscle were collected **(A)**. Total RNA was extracted from the samples and *RhoprCpt1* mRNA levels were quantified by qPCR, using *Rhopr18S* or *RhoprElf1* expression as reference gene for fat body and flight muscle samples, respectively. The results are means ± SEM, *n* = 3. (**) and (***): significantly different from dsMal by Student’s t-test at *p* < 0.01 and 0.001, respectively. **(B)** Collected fat bodies and flight muscles were subjected to the β-oxidation assay. The results are means ± SEM, *n* = 3. TG content was individually measured in the collected fat bodies **(C)** and flight muscles **(D)**. The results are means ± SD, *n* = 8. (*): significantly different from dsMal by Student’s t-test at *p* < 0.05.

Next, we employed an RNA interference method to directly target *RhoprCpt1* gene expression and try to understand its relevance for insect survival during starvation. Knockdown was effectively achieved, reducing *RhoprCpt1* expression by approximately 60% and 80% in the fat body and the flight muscle, respectively (Figure 5A). Unexpectedly, although the inhibition of *RhoprCpt1* expression was efficient, no effect on β-oxidation activity was observed in the knockdown insects compared to the control insects in either organ (Figure 5B). On the other hand, there was an increase in the TG content in the fat body of the silenced females, indicating that lipid mobilization was disturbed (Figure 5C). In the flight muscle, no difference in TG content was observed (Figure 5D).

**FIGURE 6:**
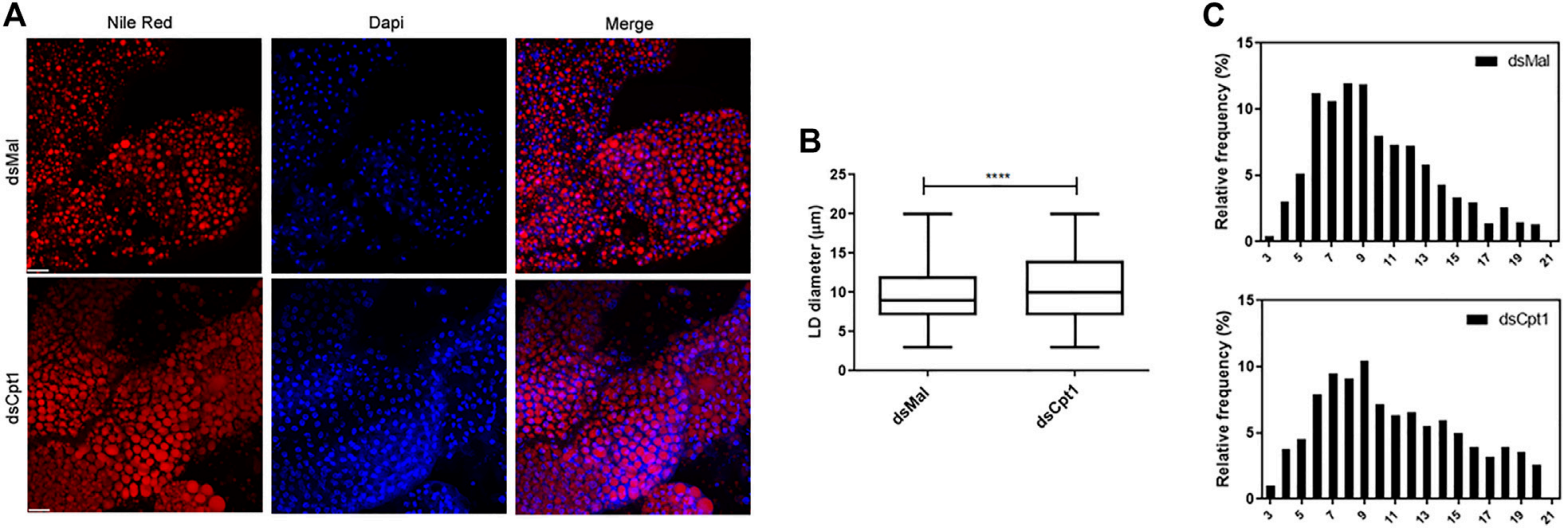
Larger lipid droplets are present in the fat bodies of *RhoprCpt1*-deficient insects. On the 10th day after the blood meal, adult females were injected with 1 µg of dsRNA for *RhoprCpt1* or *Mal* (control), were dissected 11 days later (starvation condition; day 21), and the fat bodies were collected. **(A)** After staining with Nile Red and DAPI, the fat bodies were observed under a laser scanning confocal microscope. Bars: 50 µm. **(B)** Maximum diameters of the lipid droplets (LDs) were determined from three images per group and are presented as means ± SD for at least 580 LDs per group. (****): *p* < 0.0001 by Student’s t-test. **(C)** Diameter distribution histograms of LDs from Panel **(B)**

In the fat body, as in other cells, lipids are stored in LDs, mostly in the form of TG (Walther and Farese, 2012; Toprak et al., 2020). Thus, because the TG content was higher after *RhoprCpt1* knockdown, we analyzed these organelles by fluorescence confocal microscopy. The LDs were stained with Nile Red, and their average diameters were measured. We found that they were larger in the silenced females than in the control (Figure 6A, B), in accordance with the higher TG content found in these insects. The distribution of the LDs according to their sizes (Figure 6C) showed that a higher proportion of larger lipid droplets was present in the knockdown females (10–21 µm: 54% versus 46% in the control), whereas a higher proportion of smaller LDs were present in the control females (3–9 µm: 46% versus 54% in the control).

**FIGURE 7:**
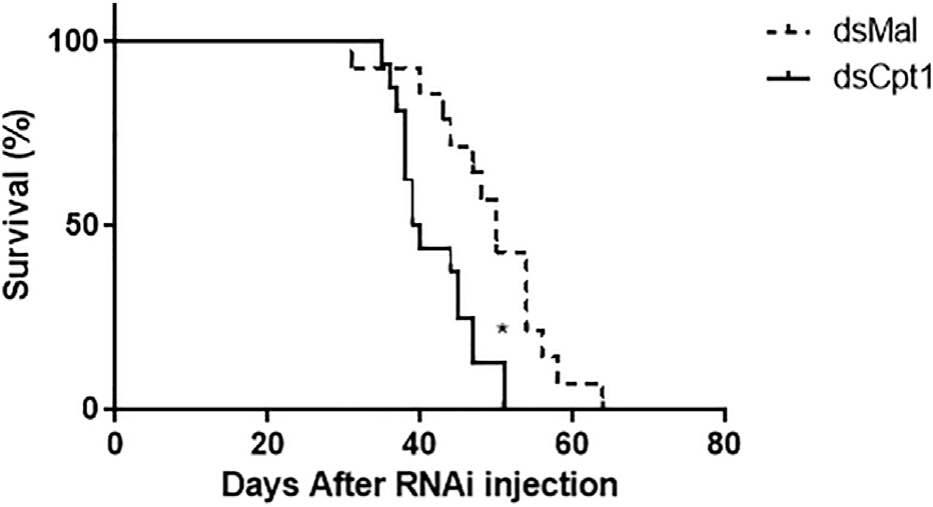
*RhoprCpt1* knockdown reduces longevity during starvation. On the 10th day after a blood meal, adult females were injected with 1 µg of dsRNA for *RhoprCpt1* or *Mal* (control), and insect mortality was monitored daily. (*): *p <* 0.05 by the log-rank test, *n* = 51–53.

As the mobilization of TG from the fat body may be critical for insect survival, the lifespan of the knockdown females was determined during starvation. The inhibition of *RhoprCpt1* gene expression affected their resistance to starvation, and the silenced females had a shorter lifespan, with a median survival time of 39.5 versus 47 days in the control group (Figure 7).

## Discussion

Stored lipids are very important for maintaining energetic homeostasis and contribute to organismal viability. When necessary, TG is mobilized from LDs, and the released fatty acids can be directed to the mitochondrial β-oxidation pathway. To enter mitochondria, activated fatty acids (acyl-CoA esters) are converted to acyl-carnitine esters, by the enzyme CPT1, and this reaction is commonly considered a critical step for the control of β-oxidation activity.

The phylogenetic analysis of the proteins with the choline/carnitine o-acyltransferase domain showed that *R. prolixus* has only one CPT1 ortholog, similar to all the other insects analyzed. This result confirms the previous findings of our group (Majerowicz et al., 2017). Moreover, this phylogenetic analysis revealed several gene duplication and loss patterns across different lineages. The presence of two independent duplications of the CPT2 gene in *R. prolixus* and *B. tabaci* suggests positive selection or functional divergence. Furthermore, extensive gene loss in the CROT clade suggests that the function of this gene may not be essential in many lineages, possibly due to redundancy with other enzymes or metabolic pathways. The diversification of the CRAT gene through multiple paralogs suggests functional divergence or neofunctionalization. These results provide a basis for generating hypotheses about the evolution and function of these enzymes and their associated domains. Further studies are needed to test these hypotheses and reveal the mechanisms driving the diversification and specialization of these enzymes across different lineages.

*RhoprCpt1* expression in the flight muscle was much higher than that in any other analyzed organ, indicating that the flight muscle probably has a high oxidative capacity, which was confirmed when β-oxidation activity was measured in the flight muscle and the fat body. Indeed, it seems that, in *R. prolixus*, lipids importantly contribute to energy generation during flight (Ward et al., 1982; Oliveira et al., 2006). These rates of β-oxidation activity during starvation were expressed as normalized per protein, and under this condition, the total protein content in the flight muscle was approximately 15 times higher than that in the fat body (∼750 µg versus ∼50 µg) (Santos-Araujo et al., 2020). Thus, we could estimate that, considering the whole organ, the capacity of the flight muscle to oxidize fatty acids can be approximately 35 times higher than that of the fat body. Although gene expression was very high in the flight muscle, it was not modulated by a blood meal, in contrast to what occurred in the fat body, where *RhoprCpt1* expression was higher in starved insects, and dramatically decreased after a blood meal. In *D. melanogaster,* the expression of diverse genes involved in lipid oxidation, including CPT1, was also higher in starved insects (Palanker et al., 2009). Surprisingly, despite the observed variation in *RhoprCpt1* gene expression, β-oxidation activity was constant before and at various days after a blood meal, indicating that the fat body of *R. prolixus* adults has a similar capacity to oxidize lipids throughout the digestive cycle.

Fatty acid oxidation was not affected by the addition of etomoxir, an inhibitor of the mammalian isoform CPT1A (Kiorpes et al., 1984), similar to what was observed in cardiac myocytes of rats chronically treated with etomoxir, where CPT1B enzymatic activity is reduced, but β-oxidation rates are not (Luiken et al., 2009). Another point to be considered is that etomoxir needs to be converted to its acyl-CoA derivative, etomoxiryl-CoA to exert its inhibitory activity, but the specific enzyme responsible for this conversion in mammals has not been identified thus far, partly due to the lack of a crystal of CPT1 with bound etomoxir (Selby and Sherratt, 1989; Rufer et al., 2009). Thus, it is not known whether insects possess the enzymatic tools required for this conversion. Additionally, etomoxir has other intracellular targets, such as interferon *γ* (INF-γ), interleukin-17 *α* **(**IL-17α), tumor necrosis factor-α (TNF-α), and pyruvate dehydrogenase (Lopaschuk et al., 1990; Mørkholt et al., 2017), showing that it is not a specific inhibitor of CPT1. The addition of malonyl-CoA also had no effect on β-oxidation rates in the fat body samples. Malonyl-CoA is produced from acetyl-CoA, by ACC. Vertebrates have two isoforms of this enzyme, ACC1 and ACC2. In mammals, ACC1 is more highly expressed in lipogenic tissues, where generated malonyl-CoA is used for lipid synthesis, and ACC2 is expressed mostly in the heart and skeletal muscle, where malonyl-CoA acts as a CPT1 inhibitor and controls fatty acid oxidation (McGarry et al., 1983; Tong, 2005). Insects and other arthropods have only one ACC isoform (Saraiva et al., 2021), and to our knowledge, no physiological effect of malonyl-CoA on the β-oxidation rate has been demonstrated. CPT1 from *D. melanogaster* was expressed in the yeast *Pichia pastoris*, and the activity of the recombinant protein was inhibited by malonyl-CoA (Jackson et al., 1999), but whether this possible CPT1 inhibition may affect the insect β-oxidation rate was not evaluated. Thus, it is still not possible to say if fatty acid oxidation is regulated by this metabolite in these animals. In *R. prolixus*, *de novo* lipid synthesis in the fat body is triggered by a blood meal (Saraiva et al., 2021), and it is therefore possible that the malonyl-CoA concentration increases after feeding, because ACC catalyzes the first step in this process, but this has not been evaluated. On the other hand, β-oxidation activity was constant before and after the blood meal, suggesting that it was not regulated by malonyl-CoA in the concentration range found in the fat body cell. Even in the mammalian heart, the effect of malonyl-CoA on the β-oxidation rate is still being discussed. This effect can also be modulated by the presence of fatty acids, since increasing concentrations of palmitoyl-CoA reduce the malonyl-CoA inhibitory capacity, as shown in mitochondria and permeabilized muscle fibers (Smith et al., 2012). The effect of malonyl-CoA on CPT1 can also be modulated by other more complex mechanisms. Hepatic CPT1A interacts with the voltage-dependent anionic channel (VDAC) and ACSL to form a complex that channels acyl-CoA toward oxidation in the mitochondria (Lee et al., 2011). As VDAC also interacts with microtubules and tubulin, alterations in the cytoskeleton of hepatocytes and other cells may affect the activity of CPT1 and its sensitivity to malonyl-CoA (Velasco et al., 1998; Miotto et al., 2017). Notably, the availability of malonyl-CoA differs between mammals, as human muscles have lower levels of this metabolite than rat muscles, despite showing the same *IC*_*50*_ values for their respective CPT1 proteins (Eaton, 2002).

It should also be considered that peroxisomes could have contributed to the measured fatty acid oxidation rates, as we did not use isolated mitochondria, and the peroxisomal enzyme acyl-CoA oxidase (ACOX) can use fatty acids of 12–18 carbons, as demonstrated in rat and human models (Osumi et al., 1980; Chu et al., 1995). However, peroxisomes do not always fully oxidize fatty acids, instead generating 8- to 12-carbon fatty acids that are then exported to the mitochondria for further oxidation (Houten et al., 2020). Whereas mitochondrial β-oxidation requires carnitine for the import of fatty acids *via* CPT1, the transport of fatty acids into peroxisomes in *D. melanogaster,* and likely in *R. prolixus* as well, is mediated by the ATP-binding cassette transporters (ABC) of subfamily D (ABCD) and is not dependent on carnitine (Tawbeh et al., 2021). The fact that the addition of carnitine significantly improved the rate of fatty acid oxidation in the fat body of starved females confirmed that a large part of the oxidation we determined in our assays likely resulted from mitochondrial β-oxidation.

The higher TG content detected in the fat bodies of *RhoprCpt1* knockdown females was also observed in *D. melanogaster* adults*,* when the gene expression of splicing factors that act on the CPT1 gene product was altered (Gingras et al., 2014; Bennick et al., 2019). The retention of TG in the silenced females was confirmed by the increase in the average diameter of the LDs and their size distribution, where a higher proportion of larger LDs was found in these insects, than in the controls. In insects, as in any other organism, lipids stored in LDs need to be mobilized to be oxidized, and both lipolysis and lipophagy may take part in this process (Zechner et al., 2012; Cingolani and Czaja, 2016; Lu et al., 2018; Santos-Araujo et al., 2020). In *D. melanogaster*, during starvation, the nuclear receptor hepatocyte nuclear factor 4 (HNF4) is activated by fatty acids freed from TG and induces the expression of enzymes involved in both lipid mobilization and β-oxidation for ATP production (Palanker et al., 2009). In line with these results, in *Aedes aegypti*, the knockdown of the HNF4 gene resulted in TG accumulation. In these mosquitoes, the expression of this nuclear receptor is activated by ecdysone, and the silencing of the ecdysone receptor causes a lower CPT1 gene expression level as well as larger LDs and a higher TG content (Wang et al., 2017). This link between lipid mobilization and oxidation was also noted when HADHA, an enzyme that takes part in the β-oxidation pathway as a component of the MTP, was silenced, and TG accumulated in the fat body of adults of *R. prolixus* (Arêdes et al., 2022). Additionally, similar to what is observed after *RhoprCpt1* knockdown, the silencing of HADHA had no impact on flight muscle TG content but led to a reduced forced flight time when compared with control insects (Arêdes et al.*,* 2022). It is noteworthy that despite the observed retention of TG in the fat body of the knockdown females and the decrease in survival during starvation, we were not able to detect any difference in β-oxidation activity. In mammals, fatty acid oxidation decreases under high levels of exercise despite a lack of increase in malonyl-CoA levels in both human and rat muscle, showing that CPT1 may not be the sole point of control of fatty acid oxidation in this tissue (Eaton, 2002). Therefore, perhaps the difference was too small in magnitude for detection, or some regulatory event acts to restore the oxidation flux. One possibility is that the fatty acid oxidation rates remained unchanged due to some unknown compensatory mechanism, such as the activity of CRAT, identified in the *R. prolixus* genome, which would generate acylcarnitine moieties in the absence of CPT1. In CRAT-knockout models, there is an increase in long-chain acylcarnitines, an indicator of incomplete β-oxidation (Muoio et al., 2012). Of course, these possibilities are highly speculative, and more studies are necessary to clarify this matter. On the other hand, we have previously shown that fatty acid oxidation is altered when other genes are silenced in *R. prolixus*. In adult females, ACSL2 knockdown results in a decrease in the β-oxidation rate, indicating that this enzyme activates fatty acids directed to this pathway (Alves-Bezerra et al., 2016). In contrast, when TG synthesis is inhibited due to GPAT1 silencing, β-oxidation is stimulated, probably because fatty acids are not used by the phosphatidic acid pathway at the same rate as in the control insects (Alves-Bezerra et al., 2017).

The reduced survival of the knockdown females under starvation conditions was similar to what is observed in the *D. melanogaster HNF4* mutants, which show lower expression of the CPT1 gene and compromised TG mobilization (Palanker et al., 2009). In contrast to these results, when HADHA was silenced in adult females of *R. prolixus,* there was no difference in lifespan, possibly because the oxidation of other substrates, such as amino acids, counterbalanced this deficiency (Arêdes et al., 2022). Moreover, we cannot discard the possibility that other factors, in addition to energetic homeostasis, may also affect the response to starvation in the knockdown insects. In *D. melanogaster*, for instance, *whitered* (*whd*) CPT1 knockout mutants are very sensitive not only to starvation, but also to oxidative stress (Strub et al., 2008). Thus, despite the very different feeding habits of these two species, it is possible that the silenced *R. prolixus* adults have a shorter lifespan not only due to a deficiency in lipid utilization but also because of higher sensitivity to the oxidative stress regularly generated by insect metabolism. As RhoprCPT1 knockdown had a relatively small impact on the survival of the fasted insects, it did not significantly affect the vectorial capacity of the kissing bug. Thus, there are better targets than RhoprCPT1 for vector population control. We cannot rule out the possibility that RhoprCPT1 knockdown affects the parasite-vector interaction, but this possibility still needs to be investigated.

*R. prolixus* is an obligatory hematophagous insect during its whole life cycle. Blood is mainly composed of proteins, and carbohydrates and lipids are present in much lower amounts (Lynch et al., 2017). These insects feed infrequently on large meals, and amino acids released from the digestion of blood proteins are thus used for the *de novo* synthesis of lipids, which are stored in the fat body to be used until the next meal (Pontes et al., 2008; Saraiva et al., 2021; Moraes et al., 2022). Thus, lipids play a central role in the metabolism of this insect. This study confirmed that the dynamics of lipid mobilization from previously synthesized stores and their efficient oxidation are essential for *R. prolixus* survival.

## Data Availability

The datasets presented in this study can be found in online repositories. The names of the repository/repositories and accession number(s) can be found in the article/Supplementary Material.

## Abbreviations

ABCD, ATP-binding cassette transporters of subfamily D; ACC, acetyl-CoA carboxylase; ACOX, acyl-CoA oxidase; ChAT, choline o-acetyltransferase; ACS, acyl-CoA synthetase; ACSL, long-chain acyl-CoA synthetase; CPT-1, carnitine palmitoyl transferase I; CPT-2, carnitine palmitoyl transferase II; CRAT, carnitine acetyltransferase; CROT, carnitine octanoyltransferase; HADHA, hydroxyacyl-CoA dehydrogenase trifunctional multienzyme complex subunit alpha; LCAD, long-chain acyl-CoA dehydrogenase; LD, lipid droplet; MCAD, medium-chain acyl-CoA dehydrogenase; MTP, mitochondrial trifunctional protein; TG, triacylglycerol; VDAC, voltage-dependent anionic channel; VLCAD, very long-chain acyl-CoA dehydrogenase.

## References

[B1] Adams M. D. Celniker S. E. Holt R. A. Evans C. A. Gocayne J. D. Amanatides P. G. (2000). The genome sequence of *Drosophila melanogaster* . Drosophila melanogaster. Sci. 287 (5461), 2185–2195. 10.1126/science.287.5461.2185 10731132

[B2] Alves-Bezerra M. Klett E. L. De Paula I. F. Ramos I. B. Coleman R. A. Gondim K. C. (2016). Long-chain acyl-CoA synthetase 2 knockdown leads to decreased fatty acid oxidation in fat body and reduced reproductive capacity in the insect *Rhodnius prolixus* . Rhodnius Prolixus. Biochim. Biophys. Acta (BBA) - Mol. Cell Biol. Lipids 1861, 650–662. 10.1016/j.bbalip.2016.04.007 PMC6421851 27091636

[B4] Alves-Bezerra M. Ramos I. B. De Paula I. F. Maya-Monteiro C. Klett E. L. Coleman R. A. (2017). Deficiency of glycerol-3-phosphate acyltransferase 1 decreases triacylglycerol storage and induces fatty acid oxidation in insect fat body. Biochim. Biophys. Acta (BBA) - Mol. Cell Biol. Lipids 1862, 324–336. 10.1016/j.bbalip.2016.12.004 PMC8826750 27956137

[B5] Ameer F. Scandiuzzi L. Hasnain S. Kalbacher H. Zaidi N. (2014). De novo lipogenesis in health and disease. Metabolism 63, 895–902. 10.1016/j.metabol.2014.04.003 24814684

[B6] Antinori S. Galimberti L. Bianco L. Grande R. Galli M. Corbellino M. S. (2017). Chagas disease in europe: A review for the internist in the globalized world. Eur. J. Intern Med. 43, 6–15. 10.1016/j.ejim.2017.05.001 28502864

[B7] Arêdes D. S. De Paula I. F. Santos-Araujo S. Gondim K. C. (2022). Silencing of mitochondrial trifunctional protein A subunit (HADHA) increases lipid stores, and reduces oviposition and flight capacity in the vector insect *Rhodnius prolixus* . Front. Insect Sci. 2. 10.3389/finsc.2022.885172 PMC10926480 38468769

[B8] Bennick R. A. Nagengast A. A. DiAngelo J. R. (2019). The SR proteins SF2 and RBP1 regulate triglyceride storage in the fat body of Drosophila. Biochem. Biophys. Res. Commun. 516 (3), 928–933. 10.1016/j.bbrc.2019.06.151 31277943

[B9] Bligh E. G. Dyer W. J. (1959). A rapid method of total lipid extraction and purification. Can. J. Biochem. Physiol. 37, 911–917. 10.1139/o59-099 13671378

[B10] Burns M. J. Nixon G. J. Foy C. A. Harris N. (2005). Standardisation of data from real-time quantitative PCR methods - evaluation of outliers and comparison of calibration curves. BMC Biotechnol. 5, 31. 10.1186/1472-6750-5-31 16336641 PMC1326201

[B11] Bustin S. A. Benes V. Garson J. A. Hellemans J. Huggett J. Kubista M. (2009). The MIQE guidelines: Minimum information for publication of quantitative real-time PCR experiments. Clin. Chem. 55, 611–622. 10.1373/clinchem.2008.112797 19246619

[B12] Carter H. E. Bhattacharyya P. K. Weidman K. R. Fraenkel G. (1952). Chemical studies on vitamin BT isolation and characterization as carnitine. Arch. Biochem. Biophys. 38, 405–416. 10.1016/0003-9861(52)90047-7 12997117

[B13] Chen W. Wosula E. N. Hasegawa D. K. Casinga C. Shirima R. R. Fiaboe K. K. M. (2019). Genome of the African cassava whitefly *Bemisia tabaci* and distribution and genetic diversity of cassava-colonizing whiteflies in Africa. Insect biochem. Mol. Biol. 110, 112–120. 10.1016/j.ibmb.2019.05.003 31102651

[B14] Chu R. Varanasi U. Chu S. Lin Y. Usuda N. Rao M. S. (1995). Overexpression and characterization of the human peroxisomal acyl-CoA oxidase in insect cells. J. Biol. Chem. 270 (9), 4908–4915. 10.1074/jbc.270.9.4908 7876265

[B15] Cingolani F. Czaja M. J. (2016). Regulation and functions of autophagic lipolysis. Trends Endocrinol. Metab. 10, 696–705. 10.1016/j.tem.2016.06.003 PMC5035575 27365163

[B16] Cohen I. Guillerault F. Girard J. Prip-Buus C. (2001). The N-terminal domain of rat liver carnitine palmitoyltransferase 1 contains an internal mitochondrial import signal and residues essential for folding of its C-terminal catalytic domain. J. Biol. Chem. 276 (7), 5403–5411. 10.1074/jbc.M009555200 11087756

[B17] Consortium C. S. (1998). Genome sequence of the nematode *C. elegans*: A plataform for investigating biology. Science 282 (5396), 2012–2018. 10.1126/science.282.5396.2012 9851916

[B18] Consortium H. G. (2012). Butterfly genome reveals promiscuous exchange of mimicry adaptations among species. Nature 487, 94–98. 10.1038/nature11041 22722851 PMC3398145

[B19] Daims H. Lücker S. Wagner M. (2006). Daime, a novel image analysis program for microbial ecology and biofilm research. Environ. Microbiol. 8, 200–213. 10.1111/j.1462-2920.2005.00880.x 16423009

[B20] Defferrari M. S. Orchard I. Lange A. B. (2016). Identification of the first insulin-like peptide in the disease vector *Rhodnius prolixus*: Involvement in metabolic homeostasis of lipids and carbohydrates. Insect biochem. Mol. Biol. 70, 148–159. 10.1016/j.ibmb.2015.12.009 26742603

[B21] Eaton S. (2002). Control of mitochondrial beta-oxidation flux. Prog. Lipid Res. 41 (3), 197–239. 10.1016/s0163-7827(01)00024-8 11814524

[B23] Felsenstein J. (1981). Evolutionary trees from DNA sequences: A maximum likelihood approach. J. Mol. Evol. 17, 368–376. 10.1007/BF01734359 7288891

[B24] Fraenkel G. (1948). B_t_, a new vitamin of the B-group and its relation to the folic acid group, and other anti-anaemia factors. Nature 161 (4103), 981–983. 10.1038/161981a0 18864012

[B25] Fraser F. Corstorphine C. G. Zammit V. A. (1997). Topology of carnitine palmitoyltransferase I in the mitochondrial outer membrane. Biochem. J. 323, 711–718. 10.1042/bj3230711 9169604 PMC1218374

[B26] Gingras R. M. Warren M. E. Nagengast A. A. DiAngelo J. R. (2014). The control of lipid metabolism by mRNA splicing in *Drosophila* . Biochem. Biophysical Res. Commun. 443 (2), 672–676. 10.1016/j.bbrc.2013.12.027 PMC4412844 24333419

[B28] Hansen I. A. Attardo G. M. Roy S. G. Raikhel A. S. (2005). Target of rapamycin-dependent activation of S6 kinase is a central step in the transduction of nutritional signals during egg development in a mosquito. J. Biol. Chem. 280, 20565–20572. 10.1074/jbc.M500712200 15788394

[B29] Houten S. M. Wanders R. J. A. (2010). A general introduction to the biochemistry of mitochondrial fatty acid β-oxidation. J. Inherit. Metab. Dis. 33 (5), 469–477. 10.1007/s10545-010-9061-2 20195903 PMC2950079

[B30] Houten S. M. Wanders R. J. A. Ranea-Robles P. (2020). Metabolic interactions between peroxisomes and mitochondria with a special focus on acylcarnitine metabolism. Biochim. Biophys. Acta Mol. Basis Dis. 1866 (5), 165720. 10.1016/j.bbadis.2020.165720 32057943 PMC7146961

[B31] Jackson V. N. Cameron J. M. Zammit V. A. Price N. T. (1999). Sequencing and functional expression of the malonyl-CoA-sensitive carnitine palmitoyltransferase from *Drosophila melanogaster* . Biochem. J. 341, 483–489. 10.1042/bj3410483 10417309 PMC1220383

[B34] Kinsella R. J. Kähäri A. Haider S. Zamora J. Proctor G. Spudich G. (2011). Ensembl BioMarts: A hub for data retrieval across taxonomic space. Database 2011, bar030. 10.1093/database/bar030 21785142 PMC3170168

[B35] Kiorpes T. C. Hoerr D. Ho W. Weaner L. E. Inman M. G. Tutwiler G. F. (1984). Identification of 2-tetradecylglycidyl coenzyme A as the active form of methyl 2-tetradecylglycidate (methyl palmoxirate) and its characterization as an irreversible, active site-directed inhibitor of carnitine palmitoyltransferase A in isolated rat liver mitochondria. J. Biol. Chem. 259, 9750–9755. 10.1016/S0021-9258(17)42763-3 6547720

[B36] Kirkness E. F. Haas B. J. Sun W. Braig H. R. Perotti M. A. Clark J. M. (2010). Genome sequences of the human body louse and its primary endosymbiont provide insights into the permanent parasitic lifestyle. Proc. Natl. Acad. Sci. U. S. A. 107, 12168–12173. 10.1073/pnas.1003379107 20566863 PMC2901460

[B37] Kishita Y. Tsuda M. Aigaki T. (2012). Impaired fatty acid oxidation in a *Drosophila* model of mitochondrial trifunctional protein (MTP) deficiency. Biochem. Biophysical Res. Commun. 419, 344–349. 10.1016/j.bbrc.2012.02.026 22342726

[B38] Larkin M. A. Blackshields G. Brown N. P. Chenna R. McGettigan P. A. McWilliam H. (2007). Clustal W and clustal X version 2.0. Bioinformatics 23, 2947–2948. 10.1093/bioinformatics/btm404 17846036

[B39] Lavárías S. Pasquevich M. Y. Dreon M. S. Heras H. (2009). Partial characterization of a malonyl-CoA-sensitive carnitine O-palmitoyltransferase I from *Macrobrachium borellii (Crustacea: Palaemonidae*). Comp. Biochem. Physiol. B Biochem. Mol. Biol. 152 (4), 364–369. 10.1016/j.cbpb.2009.01.004 19171199

[B40] Lee K. Kerner J. Hoppel C. L. (2011). Mitochondrial carnitine palmitoyltransferase 1a (CPT-1a) is part of an outer membrane fatty acid transfer complex. J. Biol. Chem. 286, 25655–25662. 10.1074/jbc.M111.228692 21622568 PMC3138250

[B41] Leyria J. Orchard I. Lange A. B. (2020). Transcriptomic analysis of regulatory pathways involved in female reproductive physiology of *Rhodnius prolixus* under different nutritional states. Sci. Rep. 10 (1), 11431. 10.1038/s41598-020-67932-4 32651410 PMC7351778

[B42] Livak K. J. Schmittgen T. D. (2001). Analysis of relative gene expression data using real-time quantitative PCR and the 2(-Delta Delta C(T)) Method. Methods 25 (4), 402–408. 10.1006/meth.2001.1262 11846609

[B90] Lynch S. A. Mullen A. M. O’Neill E. E. García C. A. (2017). Harnessing the potential of blood proteins as functional ingredients: a review of the state of the art in blood processing. Compr. Rev. Food Sci. Food Saf. 16 (3), 330–344. 10.1111/1541-4337.12254 33371539

[B43] Lopaschuk G. D. Spafford M. A. Davies N. Wall S. R. (1990). Glucose and palmitate oxidation in isolated working rat hearts reperfused after a period of transient global ischemia. Circ. Res. 66, 546–553. 10.1161/01.res.66.2.546 2297817

[B44] Lowry O. H. Rosebrough N. J. Farr A. L. Randall R. J. (1951). Protein measurement with the Folin phenol reagent. J. Biol. Chem. 193, 265–275. 10.1016/s0021-9258(19)52451-6 14907713

[B45] Lu K. Zhou J. Chen X. Li W. Li Y. Cheng Y. (2018). Deficiency of brummer impaires lipid mobilization and JH-mediated vitellogenesis in the Brown planthopper, *Nilaparvata lugens* . Nil. Lugens. Front. Physiol. 9, 1535. 10.3389/fphys.2018.01535 PMC6218678 30425657

[B46] Luiken J. J. Niessen H. E. Coort S. L. Hoebers N. Coumans W. A. Schwenk R. W. (2009). Etomoxir-induced partial carnitine palmitoyltransferase-I (CPT-I) inhibition *in vivo* does not alter cardiac long-chain fatty acid uptake and oxidation rates. Biochem. J. 419 (2), 447–455. 10.1042/BJ20082159 19138173

[B47] Majerowicz D. Alves-Bezerra M. Logullo R. Fonseca-de-Souza A. L. MeyerFernandes J. R. Braz G. R. (2011). Looking for reference genes for real-time quantitative PCR experiments in *Rhodnius prolixus* (Hemiptera: Reduviidae). Insect Mol. Biol. 20, 713–722. 10.1111/j.1365-2583.2011.01101.x 21929722

[B48] Majerowicz D. Calderón-Fernández G. M. Alves-Bezerra M. De Paula I. F. Cardoso L. S. Juárez M. P. (2017). Lipid metabolism in *Rhodnius prolixus*: Lessons from the genome. Gene 596, 27–44. 10.1016/j.gene.2016.09.045 27697616

[B49] McGarry J. D. Brown N. F. (1997). The mitochondrial carnitine palmitoyltransferase system. From concept to molecular analysis. Eur. J. Biochem. 244, 1–14. 10.1111/j.1432-1033.1997.00001.x 9063439

[B50] McGarry J. D. Mills S. E. Long C. S. Foster D. W. (1983). Observations on the affinity for carnitine, and malonyl-CoA sensitivity, of carnitine palmitoyltransferase I in animal and human tissues. Demonstration of the presence of malonyl-CoA in non-hepatic tissues of the rat. Biochem. J. 214, 21–28. 10.1042/bj2140021 6615466 PMC1152205

[B51] McGarry J. D. (2001). Travels with carnitine palmitoyltransferase I: From liver to germ cell with stops in between. Biochem. Soc. Trans. 29, 241–245. 10.1042/0300-5127:0290241 11356162

[B53] Mesquita R. D. Vionette-Amaral R. J. Lowenberger C. Rivera-Pomar R. Monteiro F. A. Minx P. (2015). Genome of *Rhodnius prolixus*, an insect vector of Chagas disease, reveals unique adaptations to hematophagy and parasite infection. Proc. Natl. Acad. Sci. 112, 14936–14941. 10.1073/pnas.1506226112 26627243 PMC4672799

[B54] Miotto P. M. Steinberg G. R. Holloway G. P. (2017). Controlling skeletal muscle CPT-I malonyl-CoA sensitivity: The importance of AMPK-independent regulation of intermediate filaments during exercise. Biochem. J. 474 (4), 557–569. 10.1042/BCJ20160913 27941154

[B55] Mistry J. Chuguransky S. Williams L. Qureshi M. Salazar G. A. Sonnhammer E. L. L. (2021). Pfam: The protein families database in 2021. Nucleic Acids Res. 49, D412–D419. 10.1093/nar/gkaa913 33125078 PMC7779014

[B56] Moraes B. Braz V. Santos-Araujo S. Oliveira I. A. Bomfim L. Ramos I. (2022). Deficiency of acetyl-CoA carboxylase impairs digestion, lipid synthesis, and reproduction in the kissing bug *Rhodnius prolixus* . Front. Physiol. 13, 934667. 10.3389/fphys.2022.934667 35936892 PMC9353303

[B57] Mørkholt A. S. Wiborg O. Nieland J. G. K. Nielsen S. Nieland J. D. (2017). Blocking of carnitine palmitoyl transferase 1 potently reduces stress-induced depression in rat highlighting a pivotal role of lipid metabolism. Sci. Rep. 7 (1), 2158. 10.1038/s41598-017-02343-6 28526869 PMC5438386

[B58] Muoio D. M. Noland R. C. Kovalik J. P. Seiler S. E. Davies M. N. DeBalsi K. L. (2012). Muscle-specific deletion of carnitine acetyltransferase compromises glucose tolerance and metabolic flexibility. Cell Metab. 15 (5), 764–777. 10.1016/j.cmet.2012.04.005 22560225 PMC3348515

[B59] Nurk S. Koren S. Rhie A. Rautiainen M. Bzikadze A. V. Mikheenko A. (2022). The complete sequence of a human genome. Science 376, 44–53. 10.1126/science.abj6987 35357919 PMC9186530

[B60] O'Brien D. M. Suarez R. K. (2001). Fuel use in hawkmoth (*Amphion floridensis*) flight muscle: Enzyme activities and flux rates. J. Exp. Zoology 290 (2), 108–114. 10.1002/jez.1040 11471140

[B61] Oliveira G. A. Baptista D. L. Guimarães-Motta H. Almeida I. C. Masuda H. Atella G. C. (2006). Flight-oogenesis syndrome in a blood-sucking bug: Biochemical aspects of lipid metabolism. Arch. Insect Biochem. Physiol. 62 (4), 164–175. 10.1002/arch.20132 16933278

[B62] Osumi T. Hashimoto T. Ui N. (1980). Purification and properties of acyl-CoA oxidase from rat liver. J. Biochem. 87 (6), 1735–1746. 10.1093/oxfordjournals.jbchem.a132918 7400120

[B63] Palanker L. Tennessen J. M. Lam G. Thummel C. S. (2009). *Drosophila* HNF4 regulates lipid mobilization and beta-oxidation. Cell Metab. 9 (3), 228–239. 10.1016/j.cmet.2009.01.009 19254568 PMC2673486

[B64] Pontes E. G. Leite P. Majerowicz D. Atella G. C. Gondim K. C. (2008). Dynamics of lipid accumulation by the fat body of *Rhodnius prolixus*: The involvement of lipophorin binding sites. J. Insect Physiol. 54, 790–797. 10.1016/j.jinsphys.2008.02.003 18395740

[B65] Price N. T. Jackson V. N. Müller J. Moffat K. Matthews K. L. Orton T. (2010). Alternative exon usage in the single CPT1 gene of Drosophila generates functional diversity in the kinetic properties of the enzyme: Differential expression of alternatively spliced variants in Drosophila tissues. J. Biol. Chem. 285 (11), 7857–7865. 10.1074/jbc.M109.072892 20061394 PMC2832936

[B66] Price N. Van Der Leij F. Jackson V. Corstorphine C. Thomson R. Sorensen A. (2002). A novel brain-expressed protein related to carnitine palmitoyltransferase I. Genomics 80 (4), 433–442. 10.1006/geno.2002.6845 12376098

[B67] Richards S. Gibbs R. A. Gerardo N. M. Consortium I. A. G. (2010). Genome sequence of the pea aphid *Acyrthosiphon pisum* . PLoS Biol. 8, e1000313. 10.1371/journal.pbio.1000313 20186266 PMC2826372

[B68] Richards S. Gibbs R. A. Weinstock G. M. Brown S. J. Denell R. Beeman R. W. (2008). The genome of the model beetle and pest *Tribolium castaneum* . Nature 452, 949–955. 10.1038/nature06784 18362917

[B69] Rosenfeld J. A. Reeves D. Brugler M. R. Narechania A. Simon S. Durrett R. (2016). Genome assembly and geospatial phylogenomics of the bed bug *Cimex lectularius* . Nat. Commun. 7, 10164. 10.1038/ncomms10164 26836631 PMC4740774

[B70] Rozen S. Skaletsky H. J. (2000). Primer3 on the WWW for general users and for biologist programmers. In: Krawetz S. Misener S. (Eds.) Bioinformatics methods and protocols: Methods in molecular biology. Humana Press, Totowa, pp. 365–386. 10.1385/1-59259-192-2:365 10547847

[B71] Rufer A. Thoma R. Hennig M. A. (2009). Structural insight into function and regulation of carnitine palmitoyltransferase. Cell Mol. Life Sci. 66 (15), 2489–2501. 10.1007/s00018-009-0035-1 19430727 PMC11115844

[B72] Santos-Araujo S. Bomfim L. Araripe L. O. Bruno R. Ramos I. Gondim K. C. (2020). Silencing of ATG6 and ATG8 promotes increased levels of triacylglycerol (TAG) in the fat body during prolonged starvation periods in the Chagas disease vector *Rhodnius prolixus* . Insect Biochem. Mol. Biol. 127, 103484. 10.1016/j.ibmb.2020.103484 33022370

[B73] Saraiva F. B. Alves-Bezerra M. Majerowicz D. Paes-Vieira L. Braz V. Almeida M. G. M. D. (2021). Blood meal drives de novo lipogenesis in the fat body of *Rhodnius prolixus* . Insect Biochem. Mol. Biol. 133, 103511. 10.1016/j.ibmb.2020.103511 33278628

[B74] Selby P. L. Sherratt H. S. (1989). Substituted 2-oxiranecarboxylic acids: A new group of candidate hypoglycaemic drugs. Trends Pharmacol. Sci. 10, 495–500. 10.1016/0165-6147(89)90049-7 2694542

[B75] Smith B. K. Perry C. G. Koves T. R. Wright D. C. Smith J. C. Neufer P. D. (2012). Identification of a novel malonyl-CoA IC(50) for CPT-I: Implications for predicting *in vivo* fatty acid oxidation rates. Biochem. J. 448 (1), 13–20. 10.1042/BJ20121110 22928974 PMC3863641

[B76] Stevenson E. (1968). The carnitine-independent oxidation of palmitate plus malate by moth flight-muscle mitochondria. Biochem. J. 110 (1), 105–110. 10.1042/bj1100105 5722681 PMC1187114

[B77] Strub B. R. Parkes T. L. Mukai S. T. Bahadorani S. Couthard A. B. Hall N. (2008). Mutations of the withered (*whd*) gene in *Drosophila melanogaster* confer hypersensitivity to oxidative stress and are lesions of the carnitine palmitoyltransferase I (CPT I) gene. Genome 51 (6), 409–420. 10.1139/G08-023 18521119

[B78] Tamura K. Stecher G. Kumar S. (2021). MEGA11: Molecular evolutionary genetics analysis version 11. Mol. Biol. Evol. 38, 3022–3027. 10.1093/molbev/msab120 33892491 PMC8233496

[B79] Tawbeh A. Gondcaille C. Trompier D. Savary S. (2021). Peroxisomal ABC transporters: An update. Int. J. Mol. Sci. 22 (11), 6093. 10.3390/ijms22116093 34198763 PMC8201181

[B80] Terrapon N. Li C. Robertson H. M. Ji L. Meng X. Booth W. (2014). Molecular traces of alternative social organization in a termite genome. Nat. Commun. 5, 3636. 10.1038/ncomms4636 24845553

[B81] Tong L. (2005). Acetyl-coenzyme A carboxylase: Crucial metabolic enzyme and attractive target for drug discovery. Cell. Mol. Life Sci. 62 (16), 1784–1803. 10.1007/s00018-005-5121-4 15968460 PMC11139103

[B82] Toprak U. Hegedus D. Doğan C. Güney G. (2020). A journey into the world of insect lipid metabolism. Arch. Insect Biochem. Physiol. 104 (2), e21682. 10.1002/arch.21682 32335968

[B83] Velasco G. Geelen M. J. Gomez Del Pulgar T. Guzman M. (1998). Malonyl-CoA-independent acute control of hepatic carnitine palmitoyltransferase I activity. Role of Ca2+/calmodulin-dependent protein kinase II and cytoskeletal components. J. Biol. Chem. 273, 21497–21504. 10.1074/jbc.273.34.21497 9705278

[B84] Walther T. C. Farese R. V. Jr. (2012). Lipid droplets and cellular lipid metabolism. Annu. Rev. Biochem. 81, 687–714. 10.1146/annurev-biochem-061009-102430 22524315 PMC3767414

[B85] Wang X. Hou Y. Saha T. T. Pei G. Raikhel A. S. Zou Z. (2017). Hormone and receptor interplay in the regulation of mosquito lipid metabolism. Proc. Natl. Acad. Sci. U. S. A. 114 (13), E2709–E2718. 10.1073/pnas.1619326114 28292900 PMC5380040

[B86] Ward J. P. Candy D. J. Smith S. N. (1982). Lipid storage and changes during flight by triatomine bugs (*Rhodnius prolixus* and *Triatoma infestans*). J. Insect Physiology 28 (6), 527–534. 10.1016/0022-1910(82)90033-6

[B87] Weinstock G. M. Robinson G. E. Gibbs R. A. Worley K. C. Evans J. D. Maleszka R. (2006). Insights into social insects from the genome of the honeybee *Apis mellifera* . Nature 443, 931–949. 10.1038/nature05260 17073008 PMC2048586

[B88] Yates A. D. Allen J. Amode R. M. Azov A. G. Barba M. Becerra A. (2022). Ensembl genomes 2022: An expanding genome resource for non-vertebrates. Nucleic Acids Res. 50, D996–D1003. 10.1093/nar/gkab1007 34791415 PMC8728113

[B89] Zechner R. Zimmermann R. Eichmann T. O. Kohlwein S. D. Haemmerle G. Lass A. (2012). FAT SIGNALS-lipases and lipolysis in lipid metabolism and signaling. Cell Metab. 7 (3), 279–291. 10.1016/j.cmet.2011.12.018 PMC3314979 22405066

