# Supplementary material for: Knockdown of carnitine palmitoyltransferase I (CPT1) reduces fat body lipid mobilization and resistance to starvation in the insect vector Rhodnius prolixus
Source: Front Physiol. 2023 Jul 4;14:1201670. doi: 10.3389/fphys.2023.1201670 (PMC10352773; doi:10.3389/fphys.2023.1201670)
Supplement: Supplementary file 1 [file DataSheet2.PDF]

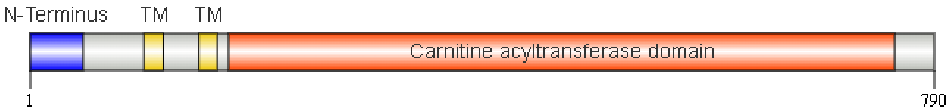

Supplementary Figure 1: RhoprCPT1 predicted protein structure. RhoprCPT1 protein structure was predicted based on searches against PFAM conserved domains database. The image was generated with IBS 2.0. N-terminus: blue; transmembrane domains (TM): yellow; carnitine acyltransferase domain: red.
