# Supplementary material for: Knockdown of carnitine palmitoyltransferase I (CPT1) reduces fat body lipid mobilization and resistance to starvation in the insect vector Rhodnius prolixus
Source: Front Physiol. 2023 Jul 4;14:1201670. doi: 10.3389/fphys.2023.1201670 (PMC10352773; doi:10.3389/fphys.2023.1201670)
Supplement: Supplementary file 2 [file DataSheet3.PDF]

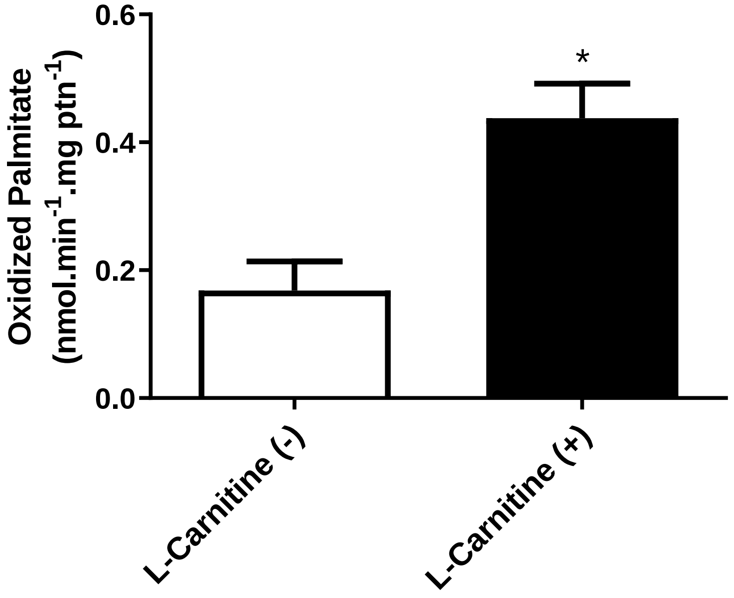

Supplementary Figure 2: Fatty acid oxidation in the fat body is stimulated by carnitine. Adult females were dissected before feeding (day 0) and their fat bodies were collected. After homogenization, the samples were used in β-oxidation assays, as described in Material and Methods, in the absence (-) or presence (+) of 100 nM L-carnitine. Results are ± means ± SEM. (*): significantly different with $p < 0.05$, by the Student's *t* test, n = 3.
