# Supplementary material for: Knockdown of carnitine palmitoyltransferase I (CPT1) reduces fat body lipid mobilization and resistance to starvation in the insect vector Rhodnius prolixus
Source: Front Physiol. 2023 Jul 4;14:1201670. doi: 10.3389/fphys.2023.1201670 (PMC10352773; doi:10.3389/fphys.2023.1201670)
Supplement: Supplementary file 3 [file DataSheet1.PDF]

Supplementary data

Supplementary Table 1: Primer sequences used in the present study.

Primer	Sequence (5' - 3')
**Primers used in qPCR reactions**	
RhoprCpt1_F	AAACACCACATGGCCAAACT
RhoprCpt1_R	GAAACGCCGTATCCATCATC
RhoprElf1_F	GATTCCACTGAACCGCCTTA
RhoprElf1_R	GCCGGGTTATATCCGATTTT
Rhopr18S_F	TCGGCCAACAAAAGTACACA
Rhopr18S_R	TGTCGGTGTAACTGGCATGT
**Primers used for dsRNA synthesis**	
RhoprCPT1_F	TAATACGACGACTCACTCACT<br>ATAGGGTGAGTTTTGCTTCCTG<br>GCTT
RhoprCPT1_R	TAATACGACGACTCACTCACT<br>ATAGGGCGCCTGAACGTCTGT<br>ATGAA
T7 minimal	TAATACGACTCACTATAGGG

F, forward
R, reverse
